# The effect of grasp compatibility in go/no-go and two-choice tasks

**DOI:** 10.3758/s13421-019-00917-5

**Published:** 2019-03-04

**Authors:** Diane Pecher, Sander Roest, René Zeelenberg

**Affiliations:** Psychology Department, Erasmus University Rotterdam, Postbus 1738, 3000 DR Rotterdam, The Netherlands

**Keywords:** Grasp compatibility, Motor affordances, Grounded cognition, Conceptual memory, Stimulus–response compatibility

## Abstract

The grasp compatibility effect has been put forward as evidence for the automatic involvement of the motor system during mental object representation. In three experiments, participants responded to object pictures or names by grasping cylinders using a precision or power grasp. In a two-choice task in which both grasps were used, we obtained grasp compatibility effects, but in a go/no-go task, in which only one grasp was used, there was no effect. These results indicate that the effect depends on the availability of response choice, in the present case, different size grasps. This suggests that grasp compatibility effects are better explained by coding of the stimulus and response on the same dimension, size, rather than automatic activation of a motor action towards the object.

When we think about a task, such as cutting a sheet of paper, we can bring to mind sensory-motor details of the experience, such as how to grasp and move the scissors, the feel of the slight resistance of the paper as the scissors cut through it, and the sound of the scissors closing. According to several views, which we will summarize as *grounded cognition*, mental representations share processing mechanisms and neural circuitry with sensory-motor processes (Barsalou, 1999; Gallese & Lakoff, 2005; Glenberg, 1997; Pulvermüller, 1999; Thill, Caligiore, Borghi, Ziemke, & Baldassarre, 2013). In this view, cognitive processes such as the activation of mental concepts rely on simulations—the reactivation of sensory-motor neural states. Evidence from behavioral and neuroscience studies indeed suggests that motor actions are a fundamental part of object conceptual knowledge and are activated automatically when manipulable objects are mentally represented (Chao, Haxby, & Martin, 1999; Chao & Martin, 2000; Creem & Profitt, 2001; Derbyshire, Ellis, & Tucker, 2006; Heard, Masson, & Bub, 2015; Masson, Bub, & Breuer, 2011; Rueschemeyer, van Rooij, Lindemann, Willems, & Bekkering, 2010; but see Pelgrims, Olivier, & Andres, 2011).

These findings suggest that information about the actions required to use an object is stored in memory and that this information is activated even when it is irrelevant for the current task. Derbyshire et al. (2006) stated that “representing the object *in what ever form* will give rise to affordance effects” (p. 95), by which they mean that affordances will be activated even if an object is retrieved from memory rather than actually present. An important finding that suggests that motor actions are activated as part of the mental representations of objects is the spatial alignment effect (Tucker & Ellis, 1998). When objects are depicted with a graspable part (e.g., a cup with a handle), manual responses are faster when handle and response side are aligned than when they are not aligned. For example, Tucker and Ellis (1998) found that spatial alignment effects between the left–right orientation of an object handle and the response location occur only when participants respond with two hands and not when they respond with different fingers of the same hand. This finding suggested that the alignment effect is due to activation of a grasping response towards the depicted object. Other studies have shown, however, that left–right alignment effects depend on several factors, such as whether attention is drawn towards the graspable part of the object by task instructions (Thomas, Stötefalk, Pecher, & Zeelenberg, 2019; Yu, Abrams, & Zacks, 2014; but see Riggio et al., 2008), whether the object handle protrudes to one side of the object (Bub & Masson, 2010; Cho & Proctor, 2011), and by the similarity of the response action to the action afforded by the stimulus (actual reach and grasp vs. key press; Bub & Masson, 2010; but see Cho & Proctor, 2013; Roest, Pecher, Naeije, & Zeelenberg, 2016). Thus, the spatial alignment effect does not provide strong evidence for the automatic activation of motor actions.

Some findings even suggest that the alignment effect is not due to overlap between stimulus and response at the motor action level, but at a more abstract level. Support for this view is the presence of alignment effects when participants responded with two fingers of the same hand (Cho & Proctor, 2010; Thomas et al., 2019) or when they responded with crossed hands or with their feet (Phillips & Ward, 2002; Thomas et al., 2019). Therefore, the alternative view, that alignment effects are due to correspondence between the stimulus and response in terms of abstract spatial codes, also seems plausible. Alignment effects may thus be a Simon-like effect. In the task context, stimulus and response may be coded in terms of task-relevant features or dimensions such as left–right orientation. Such spatial schemas are independent of modality (Chatterjee, 2010; Gentner, 2003; Gibbs, 2006) and represent space independent of specific sensory-motor features (Pecher, Boot, & van Dantzig, 2011; Pecher & Zeelenberg, 2018). Responses will be facilitated if the stimulus and response correspond on that dimension compared with when they do not (Iani, Baroni, Pellicano, & Nicoletti, 2011; Masson, 2015; Proctor & Miles, 2014). Given that several findings are hard to explain by automatic activation of motor actions towards depicted objects, one may wonder if the alignment effect can be convincingly used as evidence that motor actions are part of object representations.

One may argue that handle location (left or right side of the object) may not strongly activate action knowledge for concepts, because an object’s orientation is variant. An object’s volumetric grasp, on the other hand, is less variant (Borghi & Riggio, 2015). Because grasp size is more stable, it should be part of object knowledge. Indeed, Yu et al. (2014) argued that grip size may be activated automatically, whereas grip location may not be activated automatically, which may explain why left–right alignment effects are strongly context dependent. Consistent with the idea that grip size is activated for object concepts, several studies have shown that grasping responses are faster if they are compatible with a presented object than if they are incompatible. Tucker and Ellis (2004) presented object pictures and names in a natural/artefact decision task. Half of the objects afforded a precision grasp (e.g., screw) and the other half a power grasp (e.g., hammer). Participants responded by squeezing a response device between thumb and index finger (a precision grasp) for one category and squeezing the device with the entire hand (a power grasp) for the other category. Response times were faster if the object and response grasp were compatible than if they were incompatible. The grasp compatibility effect is associated with differences in activity in the left hemisphere within the parietal, premotor, and frontal cortex (Grezes, Tucker, Armony, Ellis, & Passingham, 2003), suggesting that the effect is due to object-related activity in neural motor areas. Grasp compatibility effects for pictures and words have been observed also in a color decision task for both volumetric and functional grasps (Bub, Masson, & Cree, 2008). That the effect is obtained for words (see also Canits, Pecher, & Zeelenberg, 2018; Gentilucci & Gangitano, 1998; Glover & Dixon, 2002; Glover, Rosenbaum, Graham, & Dixon, 2004) and for functionally related grasps supports the view that the grasp compatibility effect is due to activated conceptual action knowledge rather than visual features of the stimulus.

Just as for the spatial alignment effect, however, an alternative explanation based on abstract codes has been proposed for the grasp compatibility effect. Responses may be faster when there is correspondence in size between object and response (Masson, 2015; Proctor & Miles, 2014), which would entail that the compatibility effect is due to abstract size codes rather than direct activation of motor actions. Bub et al. (2008) tested whether the grasp compatibility effect might have been due to similarities in size or shape between the stimulus object and the response device rather than due to activation of the grasp response. Their response device consisted of four aluminum forms, each allowing a different grasp, that were arranged on a curved base in front of the participant. Participants responded to the color of object pictures by grasping or touching the form that was associated to the color. Bub et al. found that the compatibility effect was reduced to nonsignificance when participants pointed and touched the response device with the tip of their finger rather than grasped it (see also Girardi, Lindemann, & Bekkering, 2010). This finding seems problematic for the idea that correspondences in size explain the grasp compatibility effect, because it suggests that a grasping action is necessary for the compatibility effect. On the other hand, when participants responded by touching the response device they may have coded responses on a location rather than size dimension, which would have eliminated the correspondence effect.

In the present study, we aimed to test these two competing explanations of the grasp compatibility effect. If the effect is due to automatic activation of grounded action representations, the mere representation of an object should automatically activate the associated grasping action and this should influence reach and grasp responses. If, on the other hand, the grasp compatibility effect is due to correspondence of abstract modality-independent features, such as size, as has been suggested by Masson (2015) and Proctor and Miles (2014; see also Cho & Proctor, 2010), the compatibility effect is expected to occur only if size is relevant for both stimulus and response. To manipulate the relevance of grip size for the response, we used a go/no-go procedure in which participants responded with only one type of grip (either power or precision grip). In a go/no-go task, participants do not need to represent different responses, which makes the grasp size dimension irrelevant and eliminates one half of the stimulus–response correspondence (Ansorge & Wühr, 2004; Roest et al., 2016). An abstract coding account would therefore predict no grasp compatibility effect in a go/no-go task. Note, however, that according to the affordance account, the perception of an object results in the activation or potentiation of the actions afforded by the object (Tucker & Ellis, 1998). When there is a match between the actions afforded by the object and the action performed by the participant, responding is facilitated relative to situations in which there is no such match and an incongruent, competing action is activated. It has been argued that because of the automatic link between perception and action, the actions afforded by an object are activated even in the absence of action preparation or action intention. Thus, motor affordance effects should be obtained even when the task does not require the preparation of a motor response on every trial (Dixon, Goslin, & Ellis, 2012). Even on a more context-dependent view of the role of motor actions for object representations, however, motor actions should be activated if the task requires a reach and grasp response such as in the present study.

We compared the go/no-go task to a two-choice version of the task, where participants responded by grasping one of two response devices—one affording a precision grip and one affording a power grip. Note that Tucker and Ellis (2001) also used a go/no-go task, but in their version of the task a tone indicated whether the go response was precision or power grasp, thus activating two types of responses. The availability of a response alternative may activate the dimension on which they vary, creating correspondence effects (Hommel, 1996). Lien, Pedersen, and Proctor (2016) have shown that even the presence of a task-irrelevant Japanese waving cat could induce a spatial reference frame in a go/no-go auditory Simon task. Ansorge and Wühr (2004) showed that when the go/no-go task followed a two-choice task, the Simon effect still occurred, suggesting that even the memory of alternative responses induced correspondence effects. Therefore, we made sure that only one grip device was visible to participants during the go/no-go version of the task and that the go/no-go version always preceded the two-choice version. In addition, we made sure that participants had not participated in a similar experiment, which could induce the memory of a task with alternate responses.

As an exploratory analysis we also looked at the timing of the compatibility effect. The size of the effect may depend on the SOA between presentation of the object and response cue, or may differ across the RT distribution. Researchers have used these patterns to compare conditions in which the role of automatically activated actions was likely to conditions where it was unlikely (Cho & Proctor, 2010; Phillips & Ward, 2002). Interpretation of the timing of compatibility effect, however, is complicated. Some have argued that activation of afforded actions tends to occur quickly and dissipate fast, whereas effects due to conceptual processing tend to occur late or last longer (Bub, Masson, & Kumar, in press; Ferri, Riggio, Gallese, & Costantini, 2011; Hommel, 1996). Bub et al. (in press), however, observed a more or less constant effect across the RT distribution, which they interpreted as showing that the object directly activated actions because the effect occurred in the faster RTs even though it did not decrease with RT. In contrast, Derbyshire et al. (2006) have argued the opposite—namely, that effects due to activation of affordances increase with RT. Because of this mixed state of affairs, we did not have specific expectations about the timing of the grasp compatibility effect. Nevertheless, it might still be interesting to look at the distribution of compatibility effects and compare those of the two-choice task and the go/no-go task.

In Experiment 1, participants made color decisions to photographs of natural objects and artefacts. Half of the objects in each category afforded a precision grasp, and the other half afforded a power grasp. Responses were made by a reach and grasp towards one of two cylinders (Bub & Masson, 2010) that afforded a precision or a power grasp. In the go/no-go version, participants responded to one color by grasping one cylinder and withheld their response to the other color. In the two-choice version, participants responded to both colors by grasping one cylinder for one of the colors (red) and the other cylinder for the other color (blue). If the grasp compatibility effect is driven by automatic activation of motor actions towards objects, we expect that in both versions responses would be faster if the cylinder afforded the same grasp as the object. If, on the other hand, the effect is driven by correspondences in abstract codes for object size and grasp size, we expect the effect to occur only in the two-choice version of the task. Although, in principle, participants could start preparing their grasp before they see the color in the go/no-go task but not in the two-choice task, studies have shown that the degree of response preparation does not critically influence compatibility effects (Hommel, 1996).

## Experiment 1

### Method

#### Participants

Forty students at the Erasmus University Rotterdam participated for course credit.

#### Materials

**Fig. 1:**
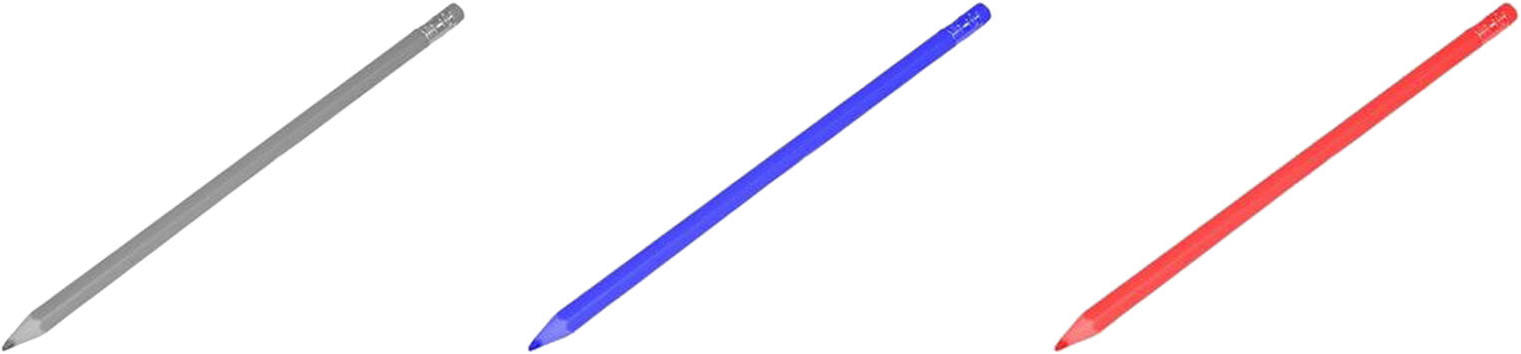
Example of a gray, blue, and red stimulus used in Experiment 1. (Color figure online)

A set of 76 black-and-white photographs were used consisting of 19 large natural objects, 19 small natural objects, 19 large artefacts, and 19 small artefacts. A red and a blue version of each photograph were created, as illustrated in Fig. 1. The complete set of items is listed in the Appendix Table 2. An additional set of 10 photographs of five natural objects and five artefacts was used for practice. Objects were shown such that they afforded a grasp with the dominant hand. Thus, for left-handed participants, the photographs were mirror images of the photographs presented to right-handed participants.

**Fig. 2:**
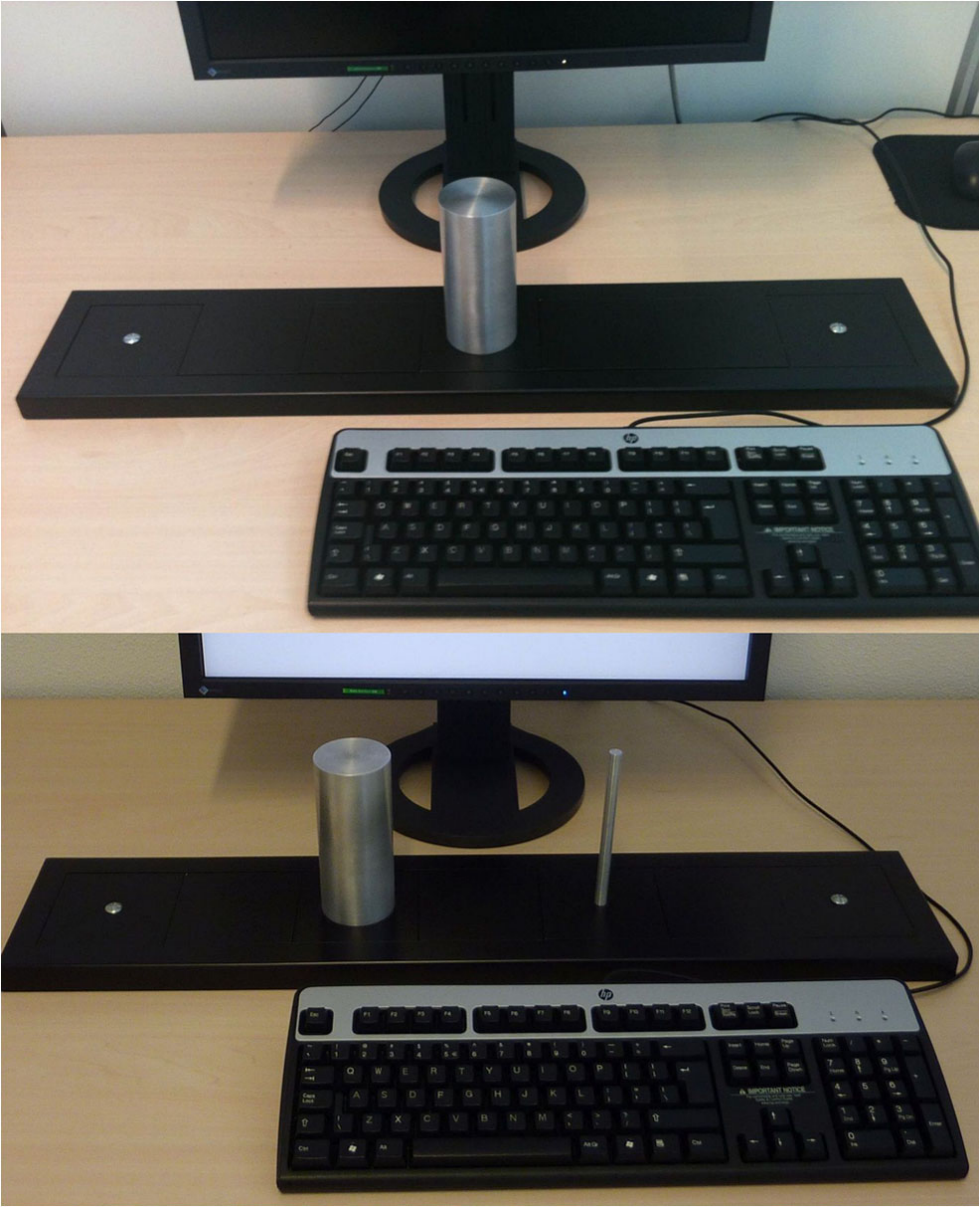
The Grabbit setup for the go/no-go version (top) and two-choice version (bottom)

A custom-made device, the Grabbit, was used to collect grasping responses (Roest et al., 2016). This device, inspired by Bub et al.’s (2008) Graspasaurus, and previously used by Roest et al. (2016) and Canits et al. (2018), consisted of a medium density fiberboard baseboard with one or two metal cylinders attached to it. The thin cylinder was 1 cm wide and afforded a precision grip (using thumb and index finger), and the thick cylinder was 6 cm wide and afforded a power grip (using the full hand). Both were 14 cm tall. During the go/no-go task, only one of the two cylinders was attached to the middle of the base, and the other cylinder was kept out of sight of the participant to prevent coding of a size reference frame. In the two-choice task, the two cylinders were placed at a distance of 20 cm from each other (from cylinder midpoint to cylinder midpoint). The cylinders were connected via an electrical wire to a Makey Makey ® (JoyLabz LLC), which translated a touch of the cylinders into key-press input to the computer. In order to close a low voltage electric circuit, the participant was also attached to the Makey Makey via a BioSemi flat electrode that was attached to their nonresponse hand. The Grabbit was placed between the computer keyboard and the computer monitor such that the *B* key on the keyboard, which was used as the starting point of the trial, was equally distant from each cylinder’s midpoint. Examples of the two setups are shown in Fig. 2.

#### Procedure

Participants were tested individually while they were seated at a desk with a computer and the Grabbit. Participants were instructed to make red/blue decisions to a series of photographs shown on the computer screen. As explained above, the experiment started with the go/no-go version of the task, followed by the two-choice version (cf. Ansorge & Wühr, 2004). Note that in a previous study using the same materials and procedure, we obtained grasp compatibility effects in a two-choice task that was not preceded by a go/no-go task (Canits et al., 2018). Thus, if we were to find a difference in compatibility effects between go/no-go and the two-choice versions, this is unlikely to have arisen from a difference in the order in which the two versions were administered. A trial started with a fixation (+ sign) in the center of the computer screen. The fixation remained on the screen until the participant pressed and held the *B* key on the computer keyboard using their dominant hand. After 250 ms, the grayscale object photograph appeared in the center of the screen. Following Bub and Masson (2010), the grayscale photograph was presented for 200 ms and was then replaced by either the red or blue version of the same photograph.

In the go/no-go task, participants responded to the go category (e.g., red) by releasing the *B* key and grasping the cylinder, and to the no-go category (e.g., blue) by holding the *B* key. Immediately after the *B* key was released or after 1,500 ms, the photograph was replaced by a blank screen. The screen remained blank until the participant had grasped the cylinder or after 1,500 ms had elapsed. If the response was incorrect, feedback (“Incorrect”) was given for 500 ms. After a correct response or after the feedback there was an interstimulus interval (ISI) of 1,000 ms until the next trial started. For the two-choice version of the task, the procedure was the same, except that participants responded to both colors by grasping one of the two cylinders using their dominant hand. The assignment of cylinder size to color (red vs blue), the assignment of color to go and no-go responses, and the position (left and right) of the cylinders in the two-choice task was counterbalanced across participants in eight versions of the experiment. In each counterbalanced version, the cylinder size for the go color was the same in the go/no-go version and the two-choice version. Both versions of the task started with 10 practice trials followed by 152 experimental trials. For the experimental trials, each photograph was presented twice. The photographs were shown in a different random order for each participant and each task version. Between the two task versions there was a short break, during which the experimenter changed the configuration of the Grabbit.

### Results

**Fig. 3:**
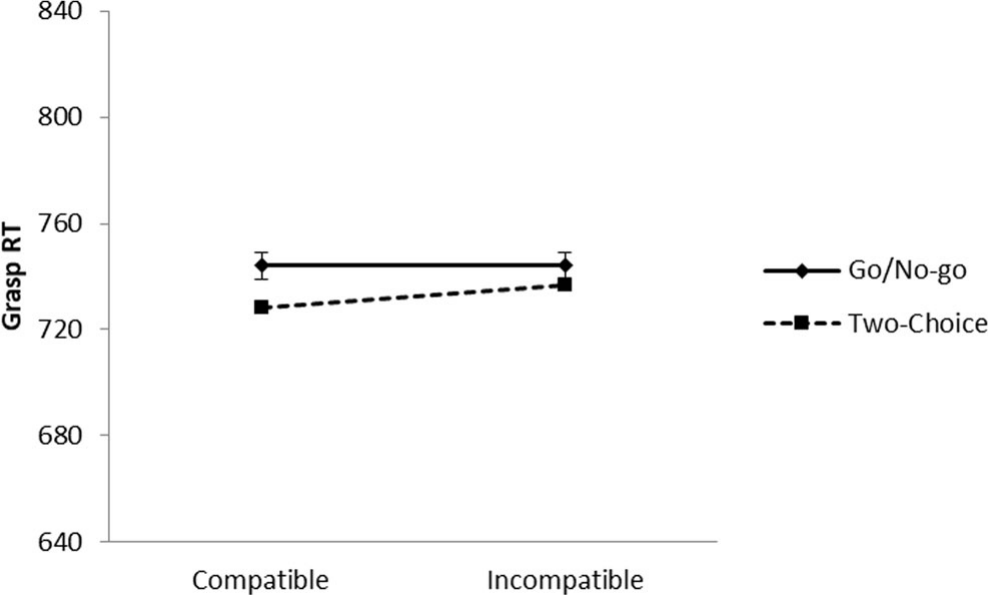
Reaction times in the color decision task to object pictures in Experiment 1. Error bars represent standard errors of the within-task difference between compatible and incompatible trials

Reaction times were measured from onset of the color picture to when the participant grasped the cylinder. Previous experiments in our lab (Canits et al., 2018) have shown compatibility effects in both movement initiation times (from picture onset to release of the *B* key) and grasping times (from key release to cylinder grasp), therefore, we looked at RT for the entire response sequence from picture onset to cylinder grasp. This is in concordance with Tucker and Ellis (2004) and Bub and Masson (2012), who also measured RT from picture onset to grasp response. Reaction times were excluded from the analyses if the response was incorrect (1.09%), the *B* key was not released between 100 and 2,000 ms after picture onset, or the cylinder was not grasped within 2,000 ms after release of the *B* key (0.30%). Mean reaction times per condition are shown in Fig. 3. All data reported in this paper, including separate release and movement times, are available on https://osf.io/x62uv/.

In addition to calculating *p* values, we calculated the JZS Bayes factor (*BF*), which is the ratio of *p*(D│H_0_) and *p*(D│H_1_), the probabilities of observing the data under the null hypothesis and the alternative hypothesis, respectively. The Bayes factor thus provides a relative measure of the extent to which the data provide evidence for the null hypothesis of no effect or the alternative hypothesis (Rouder, Speckman, Sun, Morey, & Iverson, 2009). Bayes factors between 3 and 10 can be considered moderate evidence, and Bayes factors above 10 can be considered strong evidence. Bayes factors were calculated using JASP (Love et al., 2015). Bayes factors are reported for the critical analyses—that is, the interaction between task (go/no-go task vs. two-choice task) and compatibility (compatible vs. incompatible grasp) and separate *t* tests for grasp compatibility effects in the two-choice and go/no-go tasks. The interaction was tested as a Bayesian paired-samples *t* test on the difference (between tasks) of the differences (compatible minus incompatible conditions). All Bayesian *t* tests were performed with directional hypotheses and a Cauchy prior width of 0.707.

A 2 (task) × 2 (compatibility) ANOVA indicated that reaction times did not differ between the go/no-go task and the two-choice task, *F*(1, 39) = 0.69, *p* = .412, partial η^2^ = .02. The main effect of compatibility was not significant, *F*(1, 39) = 2.82, *p* = .101, partial η^2^ = .07, and there was no interaction between task and compatibility, *F*(1, 39) = 1.84, *p* = .183, partial η^2^ = .05, *BF*_*01*_ = 1.39.^1^ Although the interaction was not significant, we performed follow-up analyses to see whether compatibility effects were present in either the go/no-go task or the two-choice task. These analyses showed that in the go/no-go task there was no effect of compatibility (mean compatibility effect = 0 ms), *t*(39) = 0.02, *p* = .982, Cohen’s *d* = 0.00, *BF*_*01*_ = 5.76, but in the two-choice task, participants responded faster to compatible than to incompatible items (mean compatibility effect = 8 ms), *t*(39) = 3.98, *p* < .001, Cohen’s *d* = 0.63, *BF*_*10*_ = 183.

**Fig. 4:**
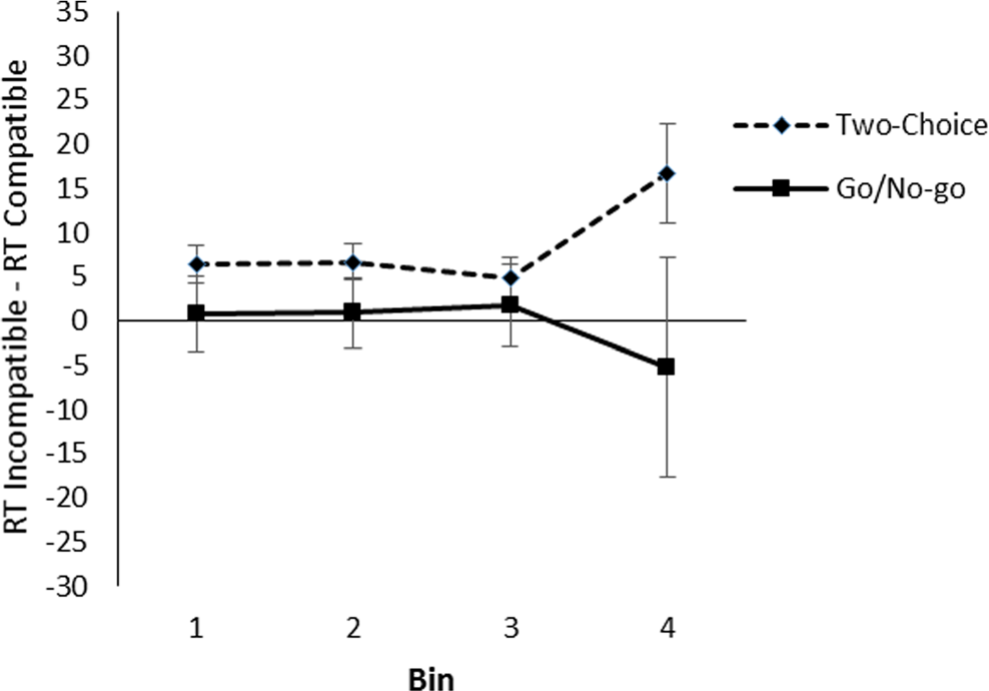
Effect of compatibility per quartile bin in the color decision task to object pictures in Experiment 1. Error bars represent standard errors of the within-bin difference between compatible and incompatible trials

To analyze the compatibility effect across the RT distribution, the valid RTs were rank ordered and divided into four bins separately for each participant and for compatible and incompatible trials such that Bin 1 contained the 25% fastest RTs, Bin 2 the next 25%, and so on (Cho & Proctor, 2010). The effect of compatibility for each bin is shown in Fig. 4. A 2 (task) × 2 (compatibility) × 4 (bin) ANOVA showed no interaction between bin and compatibility, *F*(3, 117) = 0.17, *p* = .917, partial η^2^ = .00, nor a three-way interaction between task, bin, and compatibility, *F*(3, 117) = 1.52, *p* = .213, partial η^2^ = .04. That the effect did not differ as a result of RT suggests that the difference between the tasks is not due to differences in overall RT between the tasks.

The mean error rates are shown in Table 1. As can be seen, the overall error rate was very low at 0.8%. Because of the low error rates, we expected compatibility effects to show up primarily in the RT analyses. For completeness, however, we also report statistical analyses of the error rates. A 2 (task) × 2 (compatibility) ANOVA showed a marginally significant effect of task, *F*(1, 39) = 3.31, *p* = .076, partial η^2^ = .08, which indicated that responses that slightly fewer errors were made in the go/no-go than in the two-choice task. Compatibility did not affect error rate, *F*(1, 39) = 0.10, *p* = .749, partial η^2^ = .00, and there was no interaction between task and compatibility, *F*(1, 39) = 0.01, *p* = .925, partial η^2^ = .00, *BF*_*01*_ = 5.44.

**Table 1:** Error rates (with standard errors of the mean in parentheses) in Experiments 1–3

Task	Experiment 1	Experiment 2	Experiment 3
Go/no-go			
Compatible	.003 (.002)	.035 (.019)	.067 (.029)
Incompatible	.003 (.002)	.033 (.019)	.067 (.030)
Two-choice			
Compatible	.014 (.006)	.018 (.005)	.028 (.008)
Incompatible	.013 (.005)	.029 (.005)	.037 (.009)

The results of Experiment 1 thus provided evidence for a grasp compatibility effect in the two-choice task and no effect in the go/no-go task. The absence of an interaction between task and compatibility, however, prevents us from drawing strong conclusions. In the next experiment, we investigated if more conclusive results would be found in a task in which participants made semantic decisions by responding to object category (natural or artefact), as was done in the original study by Tucker and Ellis (2004). Whereas the color decision task could be performed without much attention to the object identity, semantic decisions require object identification and therefore may lead to stronger activation of the object concept than color decisions. If motor actions are activated as part of the object representation, we should expect size compatibility effects in both tasks. If the compatibility effect is due, however, to size correspondence between stimulus and response, we should expect compatibility effects only in the two-choice task.

## Experiment 2

### Method

#### Participants

Forty students at the Erasmus University Rotterdam participated for course credit. None had participated in Experiment 1.

#### Materials and procedure

We used the same grayscale photographs as in Experiment 1. The procedure was similar to that of Experiment 1, except that objects did not change color and participants were instructed to respond to the semantic category (natural or artefact) of the object. In each category, 19 objects required a precision grip, and 19 objects required a power grip. Assignment of category to cylinder size and go or no-go condition, and assignment of cylinder to position was counterbalanced across participants.

### Results

**Fig. 5.**
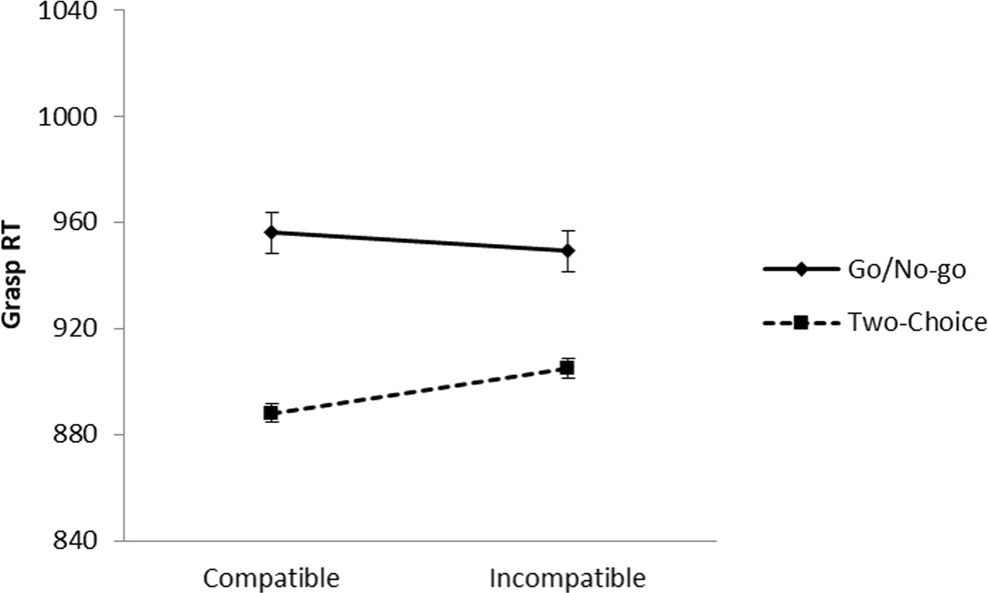
Reaction times in the artefact/natural object decision task to object pictures in Experiment 2. Error bars represent standard errors of the within-task difference between compatible and incompatible trials

Reaction times were measured from onset of the picture to when the participant grasped the cylinder. Reaction times were excluded from the analyses if the response was incorrect (3.14%), the *B* key was not released between 100 and 2,000 ms after picture onset, and the cylinder was not grasped within 2,000 ms after release of the *B* key (0.61%). Mean reaction times per condition are shown in Fig. 5. A 2 (task) × 2 (compatibility) ANOVA indicated that participants were slower in the go/no-go task than in the two-choice task, *F*(1, 39) = 8.80, *p* = .005, partial η^2^ = .18. The main effect of compatibility was not significant, *F*(1, 39) = 1.26, *p* = .269, but the interaction between task and compatibility was significant, *F*(1, 39) = 7.46, *p* = .009, partial η^2^ = .16, *BF*_*10*_ = 8.53, indicating that the size of the grasp compatibility effect was different for the go/no-go and two-choice task. Follow-up analyses showed that in the go/no-go task there was no effect of compatibility (mean compatibility effect = −8 ms), *t*(39) = 1.02, *p* = .314, Cohen’s *d* = 0.16, *BF*_*01*_ = 11.00, but in the two-choice task participants responded faster to compatible than to incompatible trials (mean compatibility effect = 17 ms), *t*(39) = 4.67, *p* < .001, Cohen’s *d* = 0.74, *BF*_*10*_ = 1,276.

**Fig. 6:**
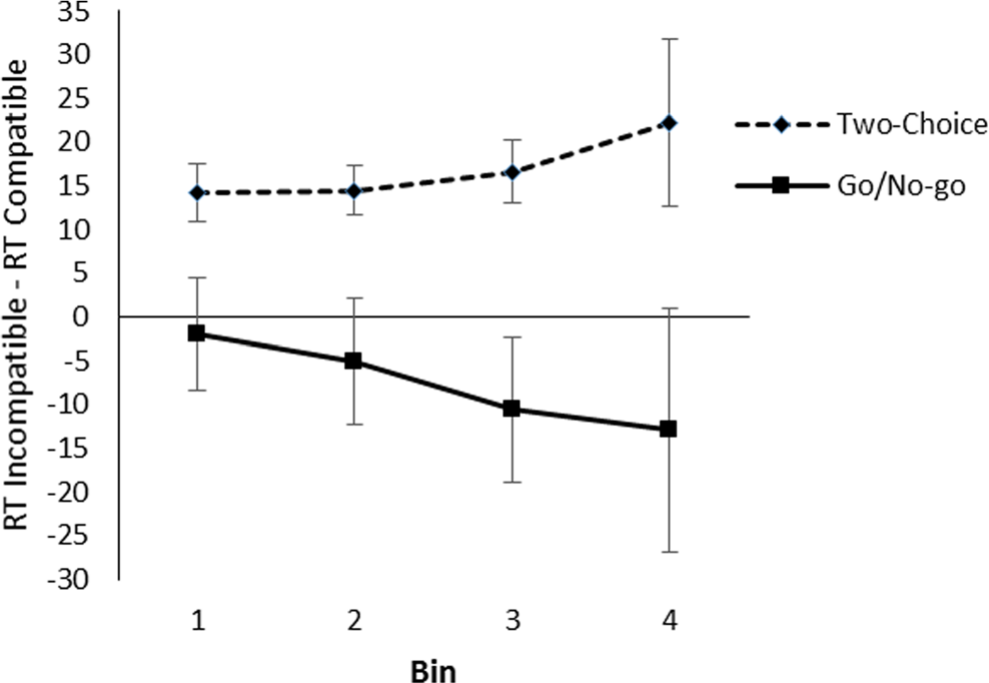
Effect of compatibility per quartile bin in the artefact/natural object decision task to object pictures in Experiment 2. Error bars represent standard errors of the within-bin difference between compatible and incompatible trials

The compatibility effect across the RT distribution is shown in Fig. 6. A 2 (task) × 2 (compatibility) × 4 (bin) ANOVA showed no interaction between bin and compatibility, *F*(3, 117) = 0.10, *p* = .959, partial η^2^ = .00, *BF*_*01*_ = 74.2, nor a three-way interaction between task, bin, and compatibility, *F*(3, 117) = 1.10, *p* = .354, partial η^2^ = .03, *BF*_*01*_ = 26.0.

The mean error rates are shown in Table 1. The ANOVA indicated that error rates did not differ between the go/no-go task and the two-choice task, *F*(1, 39) = 0.35, *p* = .556, partial η^2^ = .01. The main effect of compatibility was not significant, *F*(1, 39) = 1.96, *p* = .170, partial η^2^ = .05, and the interaction indicated that the effect of compatibility differed between tasks, *F*(1, 39) = 5.83, *p* = .021, partial η^2^ = .13, *BF*_*10*_ = 4.39. Follow-up analyses showed that in the go/no-go task there was no difference, *t*(39) = 0.60, *p* = .555, Cohen’s *d* = 0.09, *BF*_*01*_ = 8.76, but in the two-choice task participants made fewer errors on compatible than on incompatible trials, *t*(39) = 2.34, *p* = .024, Cohen’s *d* = 0.37, *BF*_*10*_ = 3.78.

To summarize, these results show an effect of grasp compatibility in the two-choice version of the task, which replicates earlier findings of grasp compatibility effects. In the go/no-go version, again, no grasp compatibility effect was obtained. Note that Canits et al. (2018, Experiment 1) obtained a similar compatibility effect (17 ms) as we did (17 ms) in a two-choice task using the same materials and procedure, but without a preceding go/no-go task. Thus, the effect in the two-choice task seems to be unaffected by task order.

As an aside, it may seem surprising that responses were faster in the two-choice than in the go/no-go task. The mechanics of the two-choice task, however, made it possible for participants to start moving by releasing the b-key before having fully decided which response to give because they could still make both grasping responses. Therefore, part of the decision time might have been absorbed in the movement time. In the go/no-go task, however, they needed to decide whether to release the *B* key or not. Therefore, to start moving before having fully decided which response to give would have resulted in incorrect responses on half of the trials. Thus, participants could initiate their response action sooner in the two-choice task than in the go/no-go task. Indeed, participants released the *B* key faster in the two-choice task (*M* = 556) than in the go/no-go task (*M* = 657). In addition, participants may also have been faster due to practice.

Next, we wanted to test if the same pattern of results could be obtained for object names. Tucker and Ellis (2004; see also Bub et al., 2008) obtained similar compatibility effects for object pictures and object names. Compatibility effects for words indicate that the effect is not entirely due to direct visual information about object affordances but, at least to some significant extent, also to object representations in memory. In Experiment 3, we therefore presented object names rather than pictures in the natural/artefact decision task.

## Experiment 3

### Method

#### Participants

Forty students at the Erasmus University Rotterdam participated for course credit. None had participated in Experiments 1 or 2.

#### Materials and procedure

Stimuli were the names of the objects used in Experiments 1 and 2. Three objects were replaced because their names were ambiguous. All stimuli are listed in the Appendix Table 2. The procedure was the same as that of Experiment 2.

### Results

**Fig. 7:**
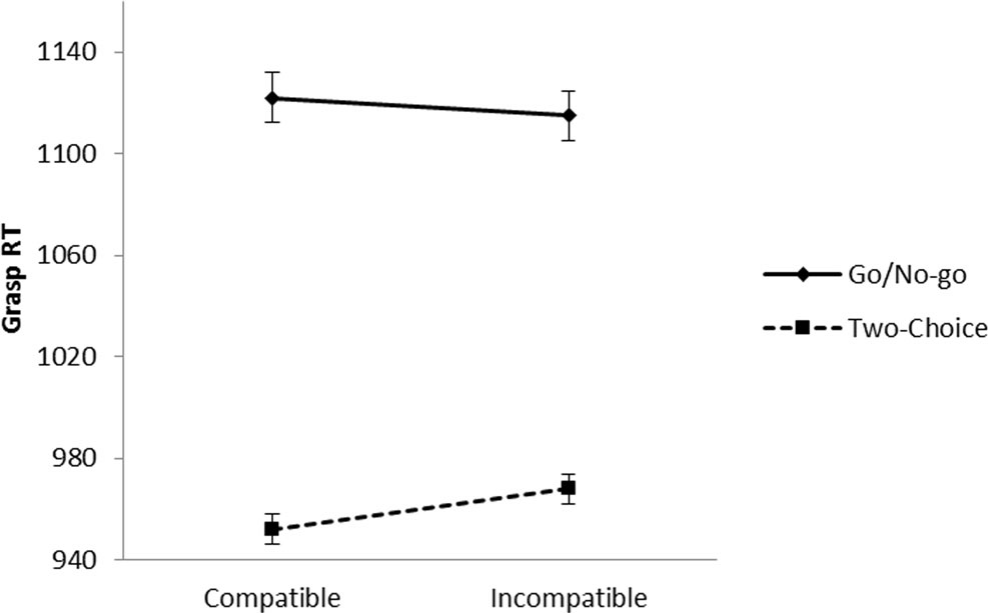
Reaction times in the artefact/natural object decision task to object names in Experiment 3. Error bars represent standard errors of the within-task difference between compatible and incompatible trials

Reaction times were measured from onset of the word to when the participant grasped the cylinder. Data from one participant were excluded because the participant had no valid reaction times in the go/no-go task, leaving data from 39 participants for the analyses. Reaction times were excluded from the analyses if the response was incorrect (5.88%), the *B* key was not released between 100 and 2,000 ms after picture onset, and the cylinder was not grasped within 2,000 ms after release of the *B* key (1.40%). Mean reaction times per condition are shown in Fig. 7. A 2 (task) × 2 (compatibility) ANOVA indicated that reaction times were slower in the go/no-go task than in the two-choice task, *F*(1, 38) = 66.36, *p* < .001, partial η^2^ = .64. The main effect of compatibility was not significant, *F*(1, 38) = 0.46, *p* = .500, partial η^2^ = .01, but the interaction indicated that the effect of compatibility was different for the go/no-go task and two-choice task, *F*(1, 38) = 4.39, *p* = .043, partial η^2^ = .10, *BF*_*10*_ = 2.39. Follow-up analyses showed that in the go/no-go task there was no effect of compatibility (mean compatibility effect = −6 ms), *t*(38) = 0.72, *p* = .476, Cohen’s *d* = 0.12, *BF*_*01*_ = 9.29, but in the two-choice task participants responded faster to compatible than to incompatible items (mean compatibility effect = 15 ms), *t*(38) = 2.57, *p* = .014, Cohen’s *d* = 0.41, *BF*_*10*_ = 6.02. Note that the compatibility effect (16 ms) in the two-choice task is similar to that of Canits et al. (2018, Experiment 2, 17 ms), who also used word names. Thus, the effect in the two-choice task again seems to be unaffected by the preceding go/no-go task.

**Fig. 8:**
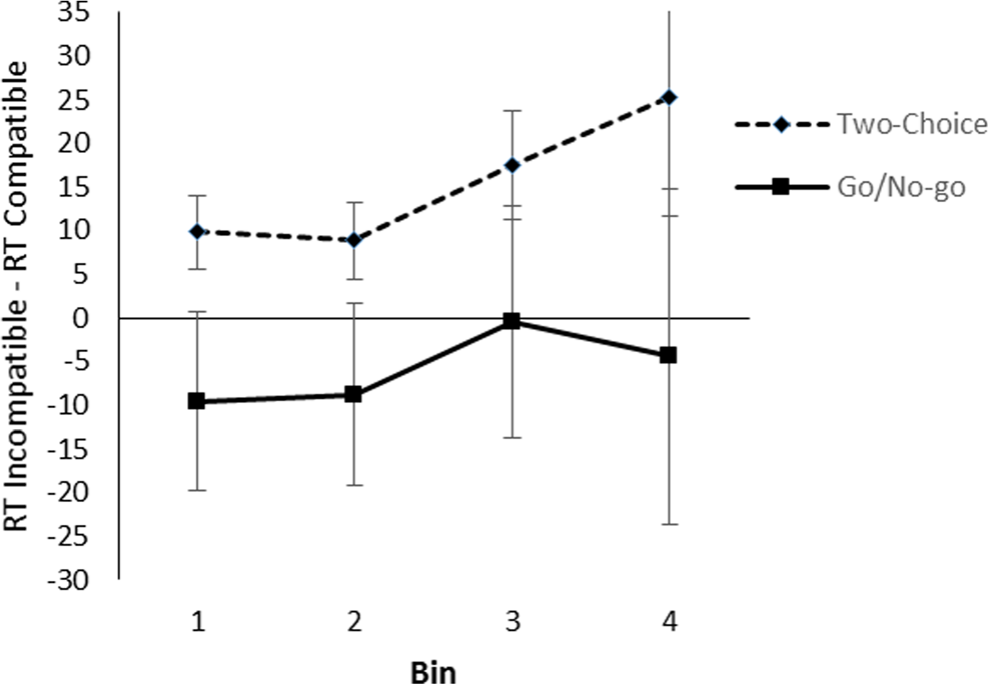
Effect of compatibility per quartile bin in the artefact/natural object decision task to object names in Experiment 3. Error bars represent standard errors of the within-bin difference between compatible and incompatible trials

The compatibility effect across the RT distribution is shown in Fig. 8. A 2 (task) × 2 (compatibility) × 4 (bin) ANOVA showed no interaction between bin and compatibility, *F*(3, 114) = 0.61, *p* = .607, partial η^2^ = .02, *BF*_*01*_ = 64.7, nor a three-way interaction between task, bin, and compatibility, *F*(3, 114) = 0.26, *p* = .857, partial η^2^ = .01, *BF*_*01*_ = 27.8.

The mean error rates are shown in Table 1. The ANOVA showed no significant effect of task, *F*(1, 38) = 0.36, *p* = .553, partial η^2^ = .01, no effect of compatibility, *F*(1, 38) = 1.62, *p* = .211, partial η^2^ = .04, and no interaction between task and compatibility, *F*(1, 38) = 2.68, *p* = .110, partial η^2^ = .07, *BF*_*10*_ = 1.10.

## Combined analyses of Experiment 1–3

To have more powerful tests of the grasp compatibility effects in the go/no-go and choice-reaction tasks, we performed additional analyses in which we combined the RT data from Experiments 1 to 3. A three-way mixed-factor ANOVA with experiment as a between-subjects factor, and task and grasp compatibility as within-subjects factors showed a significant two-way interaction between task and grasp compatibility, *F*(1, 116) = 13.23, *p* < .001, *BF*_*10*_ = 91.93, indicating that the grasp compatibility effect was larger in the two-choice task than in the go/no-go task. We subsequently performed separate two-way ANOVAs on the go/no-go task and two-choice task.

The two-way mixed ANOVA on the go/no-go task, with experiment as a between subjects factor and grasp compatibility as a within-subjects factor, showed a main effect of experiment, *F*(2, 116) = 59.15, *p* < .001. Overall responses were fastest in Experiment 1 and slowest in Experiment 3. Most important, the main effect of grasp compatibility was not significant (mean compatibility effect = −5 ms, 95% CI [−13.7 ms, 3.9 ms]), *F*(1, 116) = 1.02, *p* = .28, and neither was the interaction between grasp compatibility and experiment, *F*(1, 116) < 1, *p* = .73. A Bayesian paired-samples *t* test for the grasp compatibility effect across Experiments 1–3 provided strong evidence for the null hypothesis of no effect, *BF*_*01*_ = 19.05.

The two-way mixed ANOVA on the two-choice task, with experiment as a between subjects factor and grasp compatibility as a within-subjects factor, showed a main effect of experiment, *F*(2, 116) = 32.28, *p* < .001. Overall responses were fastest in Experiment 1 and slowest in Experiment 3. Most important, there was a significant main effect of grasp compatibility (mean compatibility effect = 13.4 ms, 95% CI [8.7 ms, 18.2 ms]), *F*(1, 116) = 31.59, *p* < .001, but no interaction between grasp compatibility and experiment, *F*(1, 116) = 1.12, *p* = .33. A Bayesian paired-samples *t* test for the grasp compatibility effect across Experiments 1–3 provided overwhelming evidence for the alternative hypothesis, *BF*_*10*_ > 100.000.^2^

## General discussion

In three experiments we examined grasp compatibility effects in a go/no-go task and a two-choice task. In all three experiments, the results showed that in the two-choice task grasp responses were facilitated if the grasp was compatible with the stimulus compared with when the grasp was incompatible with the stimulus. In contrast, no grasp compatibility effect was obtained in the go/no-go task. These results thus show that the grasp compatibility effect depends on the availability of response alternatives. When participants have to choose between two responses, the dimension on which they vary, in this case grasp size, becomes task relevant, and correspondences between stimulus and response affect performance. When participants have to choose between a grasp response and no response, however, grasp size is no longer relevant, and stimulus size does not affect performance.

Our results are in line with the view that grasp compatibility effects arise from correspondence in abstract codes between stimulus and response (Cho & Proctor, 2010; Proctor & Miles, 2014). According to this view, when stimuli and responses can be coded along the same dimension, responses are facilitated when stimulus and response on a particular trial are aligned on this dimension compared with when they are misaligned. Our results are comparable to those obtained by Ansorge and Wühr (2004), who showed that the Simon effect did not occur in a go/no-go version of a regular Simon task. They argue that in the go/no-go task, participants no longer have to refer to spatial codes in order to decide which response should be given. A spatial code is still needed to make a go response, but does not have to be distinguished from a particular other spatial code. Likewise, in our go/no-go task, participants still needed to make a grasping response on go trials, but did not need to distinguish that grasping response from another one. These findings are problematic for the view that the grasp compatibility effect is caused by stimulus-driven activation of grasping actions or affordances when objects are represented. If the representation of an object would activate a grasping action, compatible grasps should have been facilitated relative to incompatible grasps, irrespective of the presence of a choice between alternative grasps (Roest et al., 2016).

This conclusion may seem at odds with some findings that have suggested a role for motor actions. For example, some researchers have found that grasp compatibility effects are obtained only when objects are depicted within reachable space, but not when they are depicted as being beyond reach (Costantini, Ambrosini, Tieri, Sinigaglia, & Committeri, 2010; Ferri et al., 2011). These findings suggest that participants activated grasping actions. It is possible, however, that objects that are depicted as being beyond reach appear less variable in size than objects that are within reachable space. Another finding that may seem at odds is that the compatibility effect was reduced to nonsignificance when participants pointed and touched one of the four response devices that were arranged in a curved line with the tip of their finger rather than grasped it (Bub et al., 2008; Girardi, et al., 2010). Bub and Masson (2010) proposed that compatibility effects are due to actions activated by the object, but only occur when there is competition between different grasping responses. Our present results are consistent with this view, but seem hard to reconcile with the idea that object concepts automatically activate actions. Although competition between response actions may increase compatibility effects, it seems unlikely that automatically activated actions would have no effect at all in the absence of response competition. Indeed, many explanations of compatibility effects refer to competition between the activated action and response action rather than competition between two response actions. On such an account, the incompatible condition in the go/no-go task should still have induced competition between the action activated by the object and the response action and resulted in slower RTs. Thus, we cannot rule out Bub and Masson’s (2010) account, but an account based on abstract size codes seems more likely and more parsimonious.

Our results support the proposal that grasp compatibility is caused by correspondence in size between object and response (Cho & Proctor, 2010; Masson, 2015; Proctor & Miles, 2014) and are consistent with similar proposals to explain the spatial alignment effect. On this account, spatial representations are not modality specific, but at a level where visual location and response location have a shared representation. Left–right alignment effects between objects with a graspable part and a left–right response action show similar patterns of results as the Simon effect and therefore might also be better explained by correspondence between abstract spatial codes. Indeed, when objects are presented centrally, the spatial alignment effect is often not obtained unless participants’ attention is focused on the graspable part by instruction (Thomas et al., 2019; Yu et al., 2014) or when participants make reach and grasp responses (Bub & Masson, 2010). When the body of the object (instead of the entire object) is centered such that the graspable part protrudes to the left or right, alignment effects are observed even when responses are unrelated to the grasping actions, such as within-hand key presses (Cho & Proctor, 2010) or responses with the feet (Phillips & Ward, 2002). Similar to the present study, Roest et al. (2016) have shown that the spatial alignment effect is absent in a go/no-go task. Although fewer studies have investigated the grasp compatibility effect, our results suggest that this effect is also better explained by correspondence in abstract codes for size rather than the automatic activation of motor affordances. More so than the spatial alignment effect, the grasp compatibility effect was expected to provide evidence for the activation of motor affordances (Borghi & Riggio, 2015; Yu et al., 2014). Nevertheless, our findings are more consistent with an abstract coding account.

Action compatibility effects are often considered as support for the grounded cognition account (Barsalou, 1999; Glenberg, 1997). On this account, cognition shares processing mechanisms with perception and action. The idea that motor actions are automatically activated during object representations is consistent with the view that motor actions are part of those object representations. Our results, however, are not consistent with such a strong version of the grounded cognition perspective. We should note, however, that several hybrid theories of grounded cognition have been proposed in which grounded and symbolic (i.e., linguistic) representations are combined (Andrews, Vigliocco, & Vinson, 2009; Borghi & Cimatti, 2009; Dove, 2014; Durda, Buchanan, & Caron, 2009; Simmons, Hamann, Harenski, Hu, & Barsalou, 2008; Zwaan, 2014). Thus, although none of these theories have been concerned with alignment or grasp compatibility effects, theories of cognition and action that incorporate both abstract spatial codes and specific motor representations can be proposed. We certainly do not argue that specific action representations do not exist. Our results do, however, suggest that grasp compatibility effects are probably best explained by abstract spatial codes and not by the activation of highly specific action representations. Although our results strongly suggest that motor actions are not automatically activated when graspable objects are mentally represented, we do not argue that motor actions are never part of object representations. People have knowledge of the potential interactions with objects, and this knowledge may be activated as part of the representation. The question remains, however, to what extent such knowledge shares processing mechanisms with actions, and as such will manifest itself as action intentions or facilitation of reach and grasp actions that are not aimed at the object itself.

To conclude, we believe that the grasp compatibility effect is a reliable effect, but its mere presence does not necessarily indicate that the grasping actions afforded by an object are automatically activated.

## Appendix

**Table 2:** Stimulus materials used in Experiments 1–3

Large artefacts	Large natural objects	Small artefacts	Small natural objects
Bierpul (Beer Mug) Boor (Drill) Champagnefles (Champagne Bottle) Koffiepot (Coffeepot) Gewicht (Dumbbell) Gieter (Watering Can) Hamer (Hammer) Kan (Jug) Kandelaar (Candle Holder) Knoflookpers (Garlic Press) Maatbeker (Measuring Cup) Ontstopper (Plunger) Pan (Pan) Paraplu (Umbrella) Spuitbus (Spraying Can) Steelpan (Saucepan) Trekker (Squeegee) Verfroller (Paint Roller) Zaklantaarn (Flashlight)	Aardappel (Potato) Aubergine (Eggplant) Banaan (Banana) Bleekselderij (Celery) Broccoli (Broccoli) Citroen (Lemon) Courgette (Zucchini) Dennenappel (Pinecone) Houtblok (Log) Komkommer (Cucumber) Mais (Corn) Mango (Mango) Paprika (Bell Pepper) Peer (Pear) Prei (Leek) Sinaasappel (Orange) Sla (Lettuce) Tak (Branch) Ui (Onion)	Haarspeld (Hair Pin) Knoop (Button) Krijtje (Crayon) Mascara (Mascara) Nietjes (Staples) Oorbel (Earring) Paperclip (Paperclip) Pil (Pill) Pincet (Tweezers) Potlood (Pencil) Rietje (Straw) Ring (Ring) Schroef (Screw) Sigaret (Cigarette) Snoepje (Candy) Spijker (Nail) Veiligheidsspeld (Safety Pin) Wasknijper (Clothespin) Wattenstaafje (Cotton Swab)	Blad (Leaf) Braam (Blackberry) Druiven (Grapes) Eikel (Acorn) Gamba (Gamba) Knoflook (Garlic) Koffieboon (Coffee Bean) Mossel (Mussel) Olijf (Olive) Paardenbloem (Dandelion) Paddenstoel (Mushroom) Peterselie (Parsley) Pinda (Peanut) Pistache (Pistachio) Roos (Rose) Sperzieboon (Green Bean) Spruitjes (Sprouts) Veer (Feather) Walnoot (Walnut)

*Note*. Stimuli were presented as pictures in Experiments 1 and 2, and as Dutch words in Experiment 3; Approximate English translations are provided in parentheses. In Experiment 3, the following items were replaced: Gewicht (Dumbbell) ➔ Jampot (Jam Jar), Trekker (Squeegee) ➔ Zaag (Saw), and Eikel (Acorn) ➔ Doperwt (Pea)

## Footnotes

1 Throughout this paper we report *BF*_*01*_ if the evidence is in favor of *H*_*0*_, and *BF*_*10*_ if the evidence is in favor of *H*_*1*_.

2 Separate analyses of release times (the time from stimulus onset until release of the *B* key) and movement times (the time from key release until grasping of the cylinder) in these combined data showed the same pattern of results. Release times showed no main effect of compatibility, *F*(1, 116) = 0.25, *p* = .619, but there was an interaction between compatibility and task, *F*(1, 116) = 6.50, *p* = .012. Follow-up analyses showed no effect of compatibility in the go/no-go task (−5 ms), *F*(1, 116) = 1.14, *p* = .288, but a significant effect in the two-choice task (7 ms), *F*(1, 116) = 16.91, *p* < .001. Movement times were overall faster for compatible than for incompatible trials, *F*(1, 116) = 7.19, *p* = .008. The compatibility effect interacted with task, *F*(1, 116) = 9.36, *p* = .003. Follow-up analyses showed no effect of compatibility in the go/no-go task (0 ms), *F*(1, 116) = 0.03, *p* = .873 but a significant effect in the two-choice task (6 ms), *F*(1, 116) = 13.37, *p* < .001. Data from each of the experiments showed this pattern, although the effects were small and not always significant.
